# Association between celiac sprue and cryopyrin associated autoinflammatory disorders: a case report

**DOI:** 10.1186/1546-0096-5-12

**Published:** 2007-06-05

**Authors:** Marcus Shaker, Susan Edwards, Henry Chionuma, Eric Shamansky, Hal M Hoffman

**Affiliations:** 1 Dartmouth-Hitchcock Medical Center Section of Pediatric Allergy and Clinical Immunology, Lebanon, NH, USA; 2 Dartmouth-Hitchcock Medical Center Section of Pediatric Gastroenterology, Lebanon, NH, USA; 3 Dartmouth-Hitchcock Medical Center Section of General Pediatrics, Lebanon, NH, USA; 4 The University of California, San Diego School of Medicine, Division of Rheumatology, Allergy, and Immunology, La Jolla, CA, USA

## Abstract

Cryopyrin-associated diseases may be characterized by rashes, fever, and sensorineural deafness, while celiac disease may present with symptoms of malabsorption and fatigue. Arthritis is seen in both conditions. We report a young child with histologically diagnosed celiac disease and a cryopyrinopathy.

## Background

Cryopyrin-associated diseases (cryopyrinopathies) such as familial cold autoinflammatory syndrome (FCAS) and Muckle-Wells syndrome (MWS) (MIM # 120100, 191900) result from mutations in the *CIAS1 *gene [1]. Celiac disease is a polygenic disease with a strong association to specific major histocompatibility Class II antigens. An association between cryopyrinopathies and celiac sprue has not been described.

## Case presentation

We report a case of cryopyrinopathy and celiac disease in a 2 year-old Caucasian child. She presented with an 8 month history of almost daily fevers lasting 6–12 hours, occurring on average four to five times per week, with temperatures as high as 104°F. She was irritable during fevers preferring to be carried when they occurred. Associated symptoms included loose stools, periodic evanescent maculopapular rashes, episodic abdominal discomfort, an intermittent pauciarticular arthralgia of the right knee and ankle without swelling, and episodic conjunctivitis. The family did not recall any worsening of the fever or rash on exposure to cold, heat, or exercise. The history was negative for visual problems, mouth sores, morning stiffness, weakness, muscle pain, photosensitivity, or other skin rash. Growth and development were otherwise normal without any expressive language delay. Family ancestry was Irish, French, and Scandinavian. Family history was notable for celiac disease in a parental sibling but no other history of rheumatologic disease.

On examination the patient was afebrile, appeared well, and moved easily about the room. Her weight was 12.4 kg (45th percentile) and the height was 89 cm (61st percentile). Conjunctivae were not injected and the oropharynx was without lesion or exudate. Neck was supple with soft anterior mobile lymph nodes. Lung exam was without rubs and cardiac exam was normal without murmur. Abdomen was soft without organomegaly or masses. There was no clubbing or active rash, gait and station were normal. Musculoskeletal examination was normal with full range of motion of the shoulders, elbows, wrists, hips, knees, and ankles without effusions.

Evaluations included a normal complete blood count, immunoglobulins, and erythrocyte sedimentation rate during periods of fever. Urine cultures were negative, abdominal ultrasound was normal, and no infectious etiologies were identified (Table 1). Ophthalmology and audiology examinations were normal. Evaluation for celiac disease included an IgA anti-tissue transglutaminase antibody that was detected at 78 units per mL (normal < 3.9). Endoscopy was performed and a duodenal biopsy demonstrated marked villous atrophy with crypt hyperplasia and increased intraepithelial lymphocytes consistent with a diagnosis of celiac sprue. Disacharidase activity was low and consistent with untreated celiac disease. A gluten-free diet was associated with modest improvement in gastrointestinal symptoms but the fevers, rash, and arthralgias continued largely unabated. Testing for cryopyrinopathy and familial fever syndromes was performed and a disease associated CIAS1 mutation was detected [1,2]. A heterozygous G > A change in the coding sequence of exon 3 of *CIAS1 *causing replacement of the normal Valine codon (GTG) with a Methionine codon (ATG) at amino acid position 200 (Val200Met) was identified. Daily treatment with Anakinra 30 mg was associated with resolution of the rash, however arthralgia and fevers have continued.

**Table 1:** Evaluation of a toddler with recurrent fever.

WBC 13.4 K/mm^3^; 57% N, 0.6% B, 36% L, 1.9% E, 4% M
Hgb 13.1 g/dL, Hct 36.5%, MCV 76.5 Fl
ESR 14 mm/hr ; ANA negative
AST 37 U/L; ALT 27 U/L; TBILI 0.1 mg/dL
U/A Protein negative; <1 WBC/HPF; <1 RBC/HPF
TSH 2.44 uIU/mL; Free T4 1.11 ng/dL
IgG 963 mg/dL; IgA 29 mg/dL; IgM 125 mg/dL
Tissue transglutaminase IgA antibody 78.7 U/mL (nl < 3.9)
Urine VMA/HVA panel 7.1 mcg/mg Cr, 17.7 mcg/mg Cr (nl < 18.0, < 23.0)
E. Histolytic antibody screen negative
Rotavirus stool antigen negative
Toxoplasma IgG/IgM negative
Brucella IgG/IgM negative
Bartonella Henselae IgG/IgM negative
Abdominal ultrasound normal
Chest roentgenogram normal
Right knee roentgenogram normal
Disaccharides panel (endoscopy)
Lactase 3.17 umol/g/min (nl > 6.6)
Maltase 40.69 umol/g/min (nl > 40.2)
Sucrase 11.12 umol/g/min (nl > 11.3)
Palatinase 2.2 umol/g/min (nl > 2.9)
Glucoamylase 42.27 umol/g/min (nl > 25)
Duodenal endoscopic biopsy
*Marked villous atrophy with crypt hyperplasia and increased intraepithelial lymphocytes consistent with celiac disease*.
CIAS1 gene analysis positive
*A heterozygous G-->A change in the coding sequence of exon 3 identified*
MVK/HIDS gene analysis negative
TNFRSF1A gene analysis (TRAPS) negative
MEFV (FMF) gene analysis negative

## Discussion

This is the first report to our knowledge of a cryopyrinopathy in a patient with celiac disease. The Val200Met change identified in this patient has been described as a nonpenetrant mutation since it has been identified in several patients with FCAS, MWS, non-specific inflammatory diseases, and normal controls. Given the ancestry of this patient it is possible that the co-occurrence of these diseases is coincidental; however, an interaction between cryopyrin, interleukin-1β, and gliadin reactive T cells also seems plausible.

Approximately 50 *CIAS1 *mutations have been reported [3]. It has been proposed that these mutations result in a gain of function for cryopyrin resulting in increased caspase-1 activation and interleukin-1β release. This is supported by the response of FCAS and MWS patients to IL-1 targeted therapy [4,5]. However, a role for cryopyrin in the regulation of apoptosis and in the interplay between innate and adaptive immunity has also been hypothesized [6,7]. Celiac disease results from a loss of T cell tolerance to gliadin epitopes encountered in gut mucosal lymphoid tissue. The pathophysiologic connection between these disorders may relate to regulation of tolerance through innate immune responses or apoptotic pathways [8,9]. Because cryopyrin plays an important role in the production of active interleukin-1β, abnormal cryopyrin responses may promote a local inflammatory milieu that could contribute to the generation of gliadin reactive T cells.

This case highlights the spectrum of clinical presentations associated with autoinflammatory conditions. It is a reminder that although a single process is usually responsible for signs and symptoms during childhood illnesses, multiple diseases can from time to time occur in the same patient and challenge Ockham's Razor. Further studies to explore the frequency of association between these disorders may be warranted.
